# Invasive Aspergillosis Complicating Severe COVID-19: Cumulative Incidence, Risk Factors, and Outcomes Between the First Wave of the Pandemic and Subsequent Waves, According to the ECMM/ISHAM Classification and a Local Case Assessment

**DOI:** 10.3390/jof12090652

**Published:** 2026-09-01

**Authors:** Marion Blaize, Charles Gibert, Dimitri Kornblum, Sophie Demeret, Jean-Michel Constantin, Antoine Monsel, Julien Mayaux, Charles-Edouard Luyt, Alexandre Lampros, Laure Kamus, Arnaud Fekkar

**Affiliations:** 1 Parasitologie-Mycologie, Groupe Hospitalier La Pitié-Salpêtrière, Assistance Publique-Hôpitaux de Paris, 75013 Paris, France; 2 Centre d’Immunologie et des Maladies Infectieuses (Cimi-Paris), Sorbonne Université, Inserm, CNRS, 75013 Paris, France; 3 Parasitologie-Mycologie, Hôpital Saint-Antoine, Assistance Publique-Hôpitaux de Paris, 75013 Paris, France; 4 Réanimation Neurologique, Groupe Hospitalier La Pitié-Salpêtrière, Assistance Publique-Hôpitaux de Paris, 75013 Paris, France; 5 Groupe de Recherche Clinique en Anesthésie Réanimation Médecine Périopératoire (GRC29), Département d’Anesthésie-Réanimation, Groupe Hospitalier La Pitié-Salpêtrière, Assistance Publique-Hôpitaux de Paris, Sorbonne Université, 75013 Paris, France; 6 Réanimation Polyvalente, Département d’Anesthésie-Réanimation, Groupe Hospitalier La Pitié-Salpêtrière, Assistance Publique-Hôpitaux de Paris, 75013 Paris, France; 7 Immunologie-Immunopathologie-Immunothérapie (I3), UMR-S 959, Sorbonne Université, Inserm, 75013 Paris, France; 8 Centre d’Investigation Clinique Intégré en Biothérapie et Immunologie (CIC-BTi), Département Inflammation-Immunopathologie-Biothérapie (DHU I2B), Sorbonne Université, Inserm, CNRS, 75013 Paris, France; 9 Réanimation Médicale Polyvalente, Département Respiration Réanimation Réhabilitation Sommeil (R3S), Groupe Hospitalier La Pitié-Salpêtrière, Assistance Publique-Hôpitaux de Paris, 75013 Paris, France; 10 Médecine Intensive Réanimation, Institut de Cardiologie, Groupe Hospitalier La Pitié–Salpêtrière, Assistance Publique-Hôpitaux de Paris, 75013 Paris, France; 11 Institute of Cardiometabolism and Nutrition (UMRS_1166-ICAN), Sorbonne Université, Inserm, 75013 Paris, France; 12 Département de Biologie Médicale, Centre Hospitalier Universitaire Félix-Guyon, 97400 Saint-Denis, La Réunion, France; 13 Processus Infectieux en Milieu Insulaire Tropical (PIMIT) UMR 9192 CNRS-1187 Inserm-49 IRD, Université de La Réunion, 97400 Saint-Denis, La Réunion, France

**Keywords:** *Aspergillus*, molds, SARS-CoV-2, CAPA, classification criteria, corticosteroids, colonization

## Abstract

The role of severe COVID-19 as an independent risk factor for invasive aspergillosis remains debated, with reported incidences varying widely across studies. We compared the cumulative incidence, risk factors, and outcomes of invasive pulmonary mold infection (IPMI) in intensive care unit patients with confirmed COVID-19 during the first epidemic wave and subsequent waves up to March 2021 at La Pitié-Salpêtrière Hospital, Paris, France. Patients were systematically screened for fungal infections and classified according to ECMM/ISHAM criteria and a more stringent local assessment. The study included 259 patients (*n* = 171, first wave and *n* = 88, subsequent waves). Patient characteristics were similar between periods, although short-course corticosteroid therapy increased over time. Mortality rates remained unchanged. Cumulative incidence of IPMI did not differ significantly between waves, regardless of the classification system used: 12.1% versus 15.1% with ECMM/ISHAM criteria and 4.2% versus 5.8% with local assessment. IPMI was associated with solid organ transplantation and pre-existing long-term corticosteroid therapy, whereas short-course corticosteroid therapy for COVID-19 was not associated with increased risk. IPMI was strongly associated with 90-day mortality. Classification as probable COVID-19-Associated Pulmonary Aspergillosis using the ECMM/ISHAM criteria may be partly influenced by the number of diagnostic samples collected. Overall, our findings underline that the classification system substantially influences both incidence estimates and the interpretation of survival outcomes.

## 1. Introduction

During the first weeks of the COVID-19 pandemic, a substantial risk for secondary invasive pulmonary aspergillosis (IPA) was reported by several studies [1,2]. These works suggested that *Aspergillus* spp. superinfection was a worrying complication of SARS-CoV-2-related severe pneumonia, with an incidence of up to 30%, recalling the complications already described during severe influenza [2,3,4,5]. This led shortly afterward to the definition of a new clinical entity called CAPA (COVID-19-Associated Pulmonary Aspergillosis), then to the establishment of criteria and recommendations for the management of this new entity, both from a diagnostic and therapeutic point of view [6]. On the other hand, a number of additional studies indicated that the incidence of invasive aspergillosis in patients with severe COVID-19 admitted to the intensive care unit (ICU) did not exceed the low incidence usually found in ICU patients with no immunosuppressive factors and without COVID-19 [7,8]. Furthermore, although there were certain pathophysiological similarities (diffuse alveolar damage, lymphopenia) between COVID-19 and influenza, significant differences (such as neutrophil recruitment, coagulopathy or cytokine pathway activation) have been demonstrated over time [9]. This contributes to explaining the differences in clinical complications, particularly aspergillosis-related superinfections, between these two viral infections.

Several points are worth noting regarding the first year of the pandemic. The virus has evolved relatively quickly, with new variants emerging with each new wave of the pandemic. Some authors have suggested a possible association between the risk of aspergillosis and the viral variant [10]; this hypothesis remains to be confirmed. As everyone knows, the SARS-CoV-2 pandemic has caused many deaths: in France, an excess mortality in March and April 2020 (the start of the first wave in France) compared to the previous year, 2019, at the same period of the year (a 27% increase in the number of deaths) was observed. This excess mortality was less pronounced between September and December 2020 (corresponding to subsequent waves of COVID-19) (a 16% increase in deaths compared to the previous year) [11]. Furthermore, the characteristics of the affected population have also changed over the course of the pandemic waves, with national government data reporting an older hospitalized population during the second wave compared with the first [12]. The decline in excess mortality can be explained in part by the evolution of the management of severe COVID-19 since the beginning of the pandemic [13]. Thus, several factors, including the systematic use of moderate-dose corticosteroids, may have changed the incidence of invasive fungal superinfections, especially IPA. Therefore, we wondered whether changes in the profile and management of patients with severe COVID-19 between the first (March–July 2020) and subsequent (up to March 2021) epidemic waves may have impacted the incidence of IPA.

To address this question, we compared the cumulative incidence of invasive pulmonary mold infection during the first and subsequent waves. Since the criteria used to define cases of aspergillosis are crucial for classification, we also compared the epidemiological characteristics of cases according to the classification criteria used. Specifically, we compared cases diagnosed using the criteria defined by the European Confederation of Medical Mycology/International Society for Human and Animal Mycology (ECMM/ISHAM) [6] and cases diagnosed using a local clinical and mycological multiparametric assessment [7]. This is a comparative study. Part of the data has been previously published [7], briefly reported or discussed in the form of case reports [14,15] or letters [16,17].

## 2. Materials and Methods

### 2.1. Design and Patients

We performed our analysis in five ICUs at La Pitié-Salpêtrière Hospital, a 1850-bed adult tertiary care center in Paris, France. All patients with severe COVID-19 admitted to the ICUs between 1 March 2020 and 31 March 2021 who were screened for mold infections (at least one sample sent to the mycology laboratory) were included. In France, the first epidemic wave was spread out between March 2020 and the summer (we retain the date of 31 July 2020). The term “subsequent waves” was used to refer to all cases that occurred between 1 August 2020 and 31 March 2021.

Severe COVID-19 was defined as acute respiratory distress syndrome requiring admission to the ICU, combined with a positive PCR test for SARS-CoV-2 from a respiratory specimen taken within 10 days before or after ICU admission (in case of a negative SARS-CoV-2 PCR result, the diagnosis of COVID-19 was based on chest imaging strongly suggestive of COVID-19 combined with a positive IgM serology result; this situation was observed in two patients in our study). The first day of hospitalization in the ICU was defined as “Day 1” (D1).

The screening strategy for invasive pulmonary mold infection was based on respiratory and/or serum sample analysis. Samples were collected twice a week and eventually more frequently for patients who were suspected of having mechanical ventilation-associated pneumonia and/or evidence of clinical disease progression. CAPA or IPMI onset was defined as the date of the first mycological criterion contributing to the final CAPA/IPMI classification.

This study is an observational cohort that complies with the MR-004 reference methodology as defined by the French Data Protection Authority (CNIL) and is registered under the authorization number 2216650.

### 2.2. Case Definition

Two classifications were used. The first was the ECMM/ISHAM consensus criteria for defining CAPA, which classify patients into three groups: proven, probable, or possible CAPA [6]. No patients were classified as proven cases as no biopsy samples (for histopathological analysis) or autopsies were performed during the study period. The ECMM/ISHAM criteria are detailed in the Supplemental Table S1.

The second classification system used to assess the probability of COVID-19-associated mold infection was a local assessment previously described [7]. Since we had been faced with a case of pulmonary fusariosis, we opted to use the term “invasive pulmonary mold infection” (IPMI) when using these criteria, which more accurately reflects the situation and is distinguishable from CAPA, the latter of which is diagnosed using the ECMM/ISHAM consensus criteria. This local assessment approach used the European Organization for Research and Treatment of Cancer (EORTC)/Mycosis Study Group (MSG) criteria for immunocompromised patients [18], while patients with no underlying immunosuppressive factors were classified as having either a putative infection (IPMI group) or false-positive/clinically irrelevant colonization or no infection (non-IPMI group) [7]. Importantly in our classification system, all patients with one or more positive mycological test results (galactomannan antigen (GM), *Aspergillus* PCR, mold culture) were analyzed as potential cases. Patients with only positive beta-glucan results were not considered as potential cases. Results were considered false-positive/clinically irrelevant colonization in the following circumstances: single positive GM or single positive *Aspergillus* PCR or single positive mold culture from respiratory samples with further negative follow-up samples in a patient without clinical degradation. Moreover, a diagnosis of invasive pulmonary mold infection was discarded for patients with favorable outcomes despite the absence of specific antifungal treatment.

The mycological criteria are quite similar between the two classifications: the number of tests required and the types of tests (mold culture, positive galactomannan, positive *Aspergillus* PCR). There are a few minor differences between classification criteria regarding the microscopic detection of mold (as specified by the ECMM/ISHAM criteria), regarding the positivity thresholds for galactomannan, which are higher in the ECMM/ISHAM classification than in the local classification, and regarding the PCR cycle threshold, set at a maximum of 36 cycles for probable pulmonary forms according to the ECMM/ISHAM classification. The main difference lies in the presence of exclusion criteria in our local classification (described above and specified in Supplemental Table S1) that are not present in the ECMM/ISHAM classification.

### 2.3. Mycological Analysis

All respiratory samples included bronchoalveolar lavage (BAL), bronchial aspirates, and sputum. They were subjected to direct microscopic examination using two staining techniques (silver staining and Giemsa staining) and cultured on three different media: chromogenic agar BBL CHROMagar Candida (Becton Dickinson GmbH, Heidelberg, Germany) incubated for 48 h at 37 °C; Malt agar MCA (bioMérieux, Marcy-l’Etoile, France) incubated for 7 days at 25 °C; and Sabouraud Chloramphenicol Gentamicin agar (BioRad, Marnes-la-Coquette, France), incubated for a maximum of 4 weeks at 37 °C and 25 °C.

In-house real-time PCR was used for the specific detection of *Aspergillus fumigatus*, as previously described [19]. Briefly, our in-house PCR is a real-time PCR that targets a 67 bp segment of a 28S ribosomal RNA-encoding DNA. DNA extraction was performed using a 1 mL sample, either with an automated technique (EMAG, bioMérieux, Marcy-l’Etoile, France) or a manual technique (QIAamp DNA blood mini kit, QIAGEN GmbH, Hilden, Germany).The elution volume was 100 µL, and the amplification step was performed in duplicate using 5 µL of eluate per well on a QuantStudio 5 thermocycler (ThermoFisher Scientific, Waltham, MA, USA). *Aspergillus fumigatus* PCR was performed on serum samples and all types of respiratory specimens.

The galactomannan index in BAL or serum (Platelia *Aspergillus* Ag, BioRad, Marnes-la-Coquette, France) was determined according to the manufacturer’s instructions. The positive cut-off was 0.5. The detection of beta-D-glucan in serum (Fungitell, Associates of Cape Cod, East Falmouth, MA, USA) was performed according to the manufacturer’s instructions. A result was considered positive when the value was above 80 pg/mL.

### 2.4. Statistical Analysis

A comparison of the clinical characteristics of patients with COVID-19 between the first and subsequent waves was performed in the overall cohort. Analyses of the cumulative incidence of fungal complications and comparison based on fungal diagnosis were performed after excluding patients for whom available mycological data were insufficient to allow classification according to the predefined diagnostic criteria (Supplemental Table S1).

Continuous variables were not assumed to follow a normal distribution and were summarized as median and interquartile range. Univariate between-group comparisons were performed using the Mann–Whitney U test. Categorical variables were summarized as counts and percentages and compared using Fisher’s exact test.

Cumulative incidence was calculated as the number of patients classified as CAPA or IPMI divided by the number of included patients with available mycological assessment during the corresponding study period. Cumulative incidences were reported as percentages with 95% confidence intervals calculated using the exact binomial method.

To identify factors associated with fungal complications (CAPA and IPMI), multivariable logistic regression was performed. Given the limited number of events for both probable CAPA and IPMI, Firth’s penalized logistic regression was applied. Covariates were selected a priori based on clinical relevance and the number of events. For CAPA, possible CAPA cases were excluded, and the analysis compared patients with probable CAPA to patients without CAPA. The prespecified model included immunosuppression status, short-course corticosteroid therapy administered for COVID-19, and the number of BAL galactomannan determinations, to account for diagnostic sampling intensity. A sensitivity model was also performed by separating host factors into solid organ transplantation and long-term corticosteroid therapy. For IPMI, the prespecified model was restricted to host factors, including solid organ transplantation and long-term corticosteroid therapy. A sensitivity model additionally included short-course corticosteroid therapy administered for COVID-19. Results are presented as odds ratios with 95% confidence intervals and *p* values.

Ninety-day survival was analyzed from ICU admission and was performed using a Mantel–Cox log-rank test.

For all tests, a *p* value below 0.05 was considered statistically significant. Descriptive analyses were performed using GraphPad Prism 10.5.0 (GraphPad Software, Boston, MA, USA). Firth’s penalized logistic regression was performed using R4.6.0 software (R Foundation, Vienna, Austria).

The study dataset is available at https://doi.org/10.5281/zenodo.21977909.

## 3. Results

### 3.1. Patient Characteristics During First and Subsequent Waves

**Figure 1:**
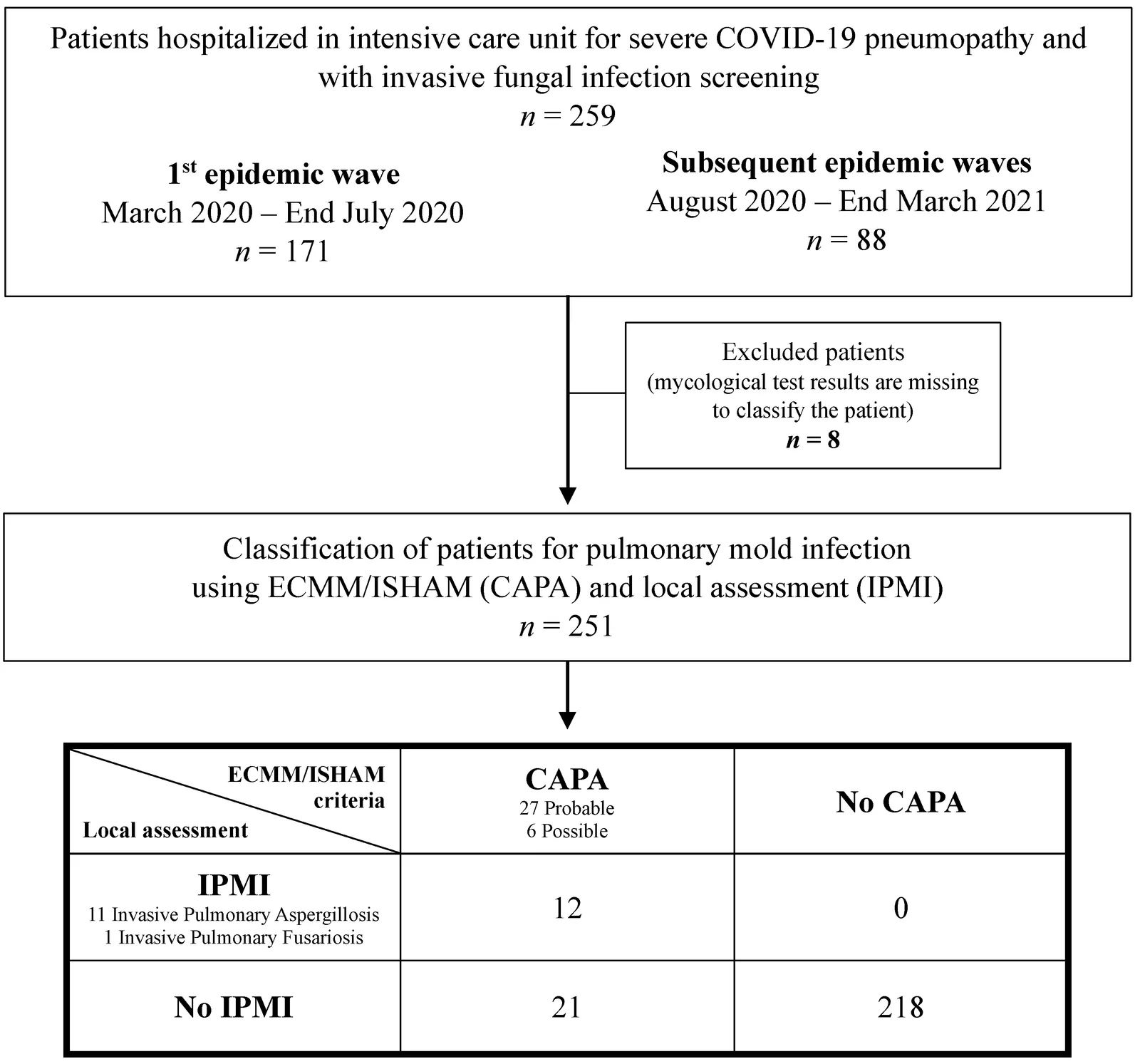
Flowchart of the study and distribution of patients according to ECMM/ISHAM classification or local assessment. ECMM/ISHAM, European Confederation for Medical Mycology/International Society for Human and Animal Mycology; CAPA, COVID-19-Associated Pulmonary Aspergillosis; IPMI, invasive pulmonary mold infection.

Overall, the study population includes 259 patients with SARS-CoV-2 infection, hospitalized in the ICU and with at least one sample sent for mycological screening (Figure 1). Demographic characteristics at ICU admission were comparable between the first wave and subsequent waves. Regarding specific COVID-19 therapies, short-term corticosteroid use increased substantially during subsequent waves (24.2% versus 92.4%, *p* < 0.001), while hydroxychloroquine and lopinavir were totally dropped (Table 1). Thus, the data indicate that patients were more severely ill during subsequent waves (median SAPS2 at ICU admission 47 versus 61, *p* > 0.001). Notably, a higher number of patients received extracorporeal membrane oxygenation (ECMO) (55.3% versus 77.8%, *p* = 0.001), while rates of pre-existing immunodepression (EORT/MSG criteria) remained equivalent (13.5% versus 10.7%, *p* = 0.688).

### 3.2. Strategy for Diagnosis of Fungal Infections and Cumulative Incidence of Aspergillosis

From the initial cohort (*n* = 259), eight patients were excluded from mycological analysis due to missing data (Figure 1).

The proportion of patients who underwent bronchoalveolar lavage was higher in subsequent waves (82.0% versus 96.5%, *p* = 0.001), with an increase in the number of BAL cultures and BAL galactomannan tests performed per patient (*p* = 0.012 and *p* = 0.0091, respectively). The number of serum samples collected per patient for diagnosing fungal infections between the first and subsequent waves was similar for all techniques used, except for a decrease in PCR targeting *A. fumigatus* (Table 2).

According to the ECMM/ISHAM criteria, a total of 27 patients were categorized as probable cases of CAPA and six were categorized as possible cases across all waves. The results indicate stability in the cumulative incidence of CAPA over time: 12.1% (20/165; 95% CI: 7.6–18.1) in the first wave and 15.1% (13/86; 95% CI: 8.3–24.5) in subsequent waves (*p* = 0.556).

Based on the local assessment, 12 patients were categorized as IPMI cases. We also found stability in the cumulative incidence of IPMI over time, 4.2% (7/165; 95% CI: 1.7–8.5) in the first wave and 5.8% (5/86; 95% CI: 1.9–13.0) in the subsequent waves (*p* = 0.551).

So, the cumulative incidence of pulmonary mold complications remained stable between the first and subsequent waves, regardless of the classification criteria.

### 3.3. Characteristics and Risk Factors Associated with Mold Infections According to Classification Strategy

In univariate analysis, patients classified as having probable CAPA or IPMI were more frequently immunocompromised than patients without fungal infection, regardless of the classification system used: 25.9% versus 10.3% of patients were immunocompromised in the CAPA and non-CAPA groups, respectively (*p* = 0.02) (ECMM/ISHAM criteria) (Table 3), and 58.3% versus 9.8% were immunocompromised in the IPMI and non-IPMI groups, respectively (*p* < 0.0001) (local criteria) (Table 4).

Using Firth’s penalized logistic regression, probable CAPA classification remained associated with immunosuppression status and BAL galactomannan sampling frequency. Immunosuppression was associated with probable CAPA with an OR of 3.95 (95% CI, 1.39–10.64; *p* = 0.011), while each additional BAL galactomannan determination was associated with increased odds of probable CAPA classification (OR, 1.27; 95% CI, 1.11–1.48; *p* < 0.001). Short-course corticosteroid therapy for COVID-19 was not associated with probable CAPA after adjustment (OR, 1.65; 95% CI, 0.69–4.03; *p* = 0.259) (Table 3). In a sensitivity model separating host factors, long-term corticosteroid therapy remained associated with probable CAPA (OR, 2.39; 95% CI, 0.79–3.92; *p* = 0.004).

Using Firth’s penalized logistic regression, solid organ transplantation and long-term corticosteroid therapy were strongly associated with IPMI. In the prespecified model restricted to host factors, solid organ transplantation was associated with IPMI with an OR of 16.41 (95% CI, 4.37–62.50; *p* < 0.001), and long-term corticosteroid therapy with an OR of 18.31 (95% CI, 2.81–99.59; *p* = 0.004) (Table 4). In a sensitivity model including short-course corticosteroid therapy for COVID-19, the associations with solid organ transplantation and long-term corticosteroid therapy remained stable, whereas short-course corticosteroid therapy was not associated with IPMI (OR, 1.00; 95% CI, 0.28–3.36; *p* = 0.999).

An unsupervised approach, using exploratory principal component analysis (PCA), revealed associations consistent with those identified in the Firth’s logistic regression analysis, although it did not completely separate the patient groups (Supplemental Figure S1).

The use of short-course corticosteroid therapy for severe COVD-19 was not independently associated with either probable CAPA or IPMI.

No significant difference in patient characteristics was observed between the IPMI group and the probable CAPA group (Supplemental Table S2). The only statistically significant finding is a higher number of *Aspergillus* PCR tests per patient in serum in the IPMI group (*p* = 0.025) (Supplemental Table S2).

### 3.4. Analysis of Observed Concordant and Discordant Cases Between the Two Diagnostic Approaches

Supplemental Table S3 provides comprehensive mycological data for the 39 patients who had at least one positive diagnostic test (culture or antigen or PCR). Local assessment and ECMM/ISHAM classifications are applied to each patient. Eighteen patients had concordant results, including the 12 patients diagnosed with IPMI (also classified as possible or probable CAPA). Discrepancies in case classification were observed in 21 patients. Thus, the diagnoses of 17 probable CAPA cases and of four possible CAPA cases were not supported by the local evaluation. Only one of these patients received antifungal therapy specifically targeted against *Aspergillus* spp.

### 3.5. Outcome and Factors Associated with Survival

**Figure 2:**
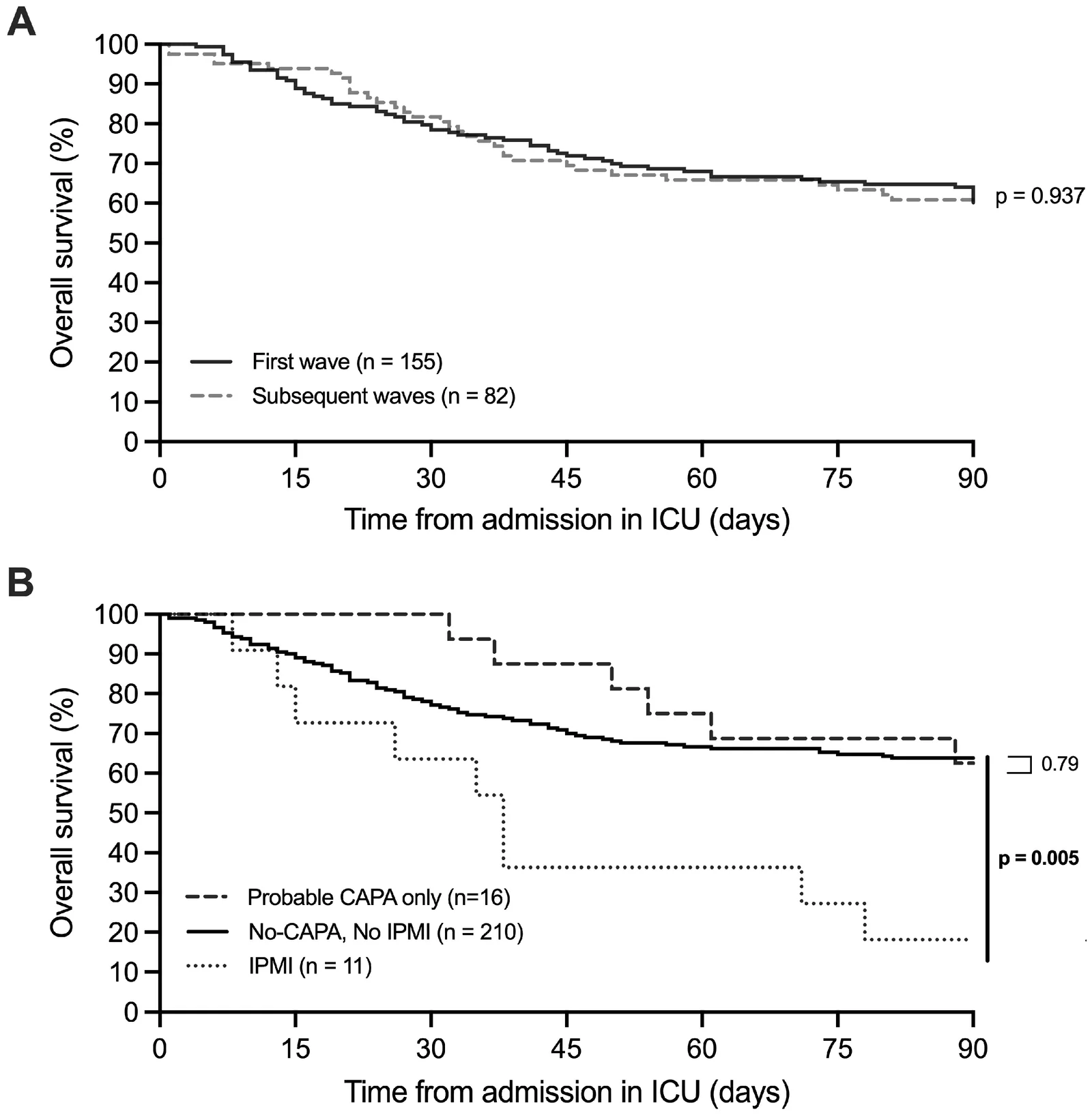
Overall survival of patients with severe COVID-19 admitted to the intensive care unit. (**A**) Outcome at day 90 after ICU admission according to the period of infection, first wave (March 2020, to end of July) or subsequent waves (August 2020, to end of March 2021). (**B**) Outcome at day 90 according to whether an episode of either CAPA or IPMI occurred. “Probable CAPA only”: patients classified as having a probable infection based on the ECMM/ISHAM criteria but not on local criteria; “IPMI”: patients classified as having an infection based on local and ECMM/ISHAM criteria (all patients categorized as IPMI were also categorized as CAPA); “no-CAPA/no-IPMI”: patients without CAPA or IPMI. *p* values of the Mantel–Cox test for the three clinical classifications are in bold. ICU, intensive care unit; CAPA, COVID-19-Associated Pulmonary Aspergillosis; IPMI: invasive pulmonary mold infection.

Overall survival at 90 days from ICU admission did not differ between the first and subsequent waves for all patients combined (Figure 2A).

We compared mortality rates among three groups: patients considered “probable CAPA only” (those not responding to IPMI classification), patients with IPMI (all corresponding also to CAPA), and patients without CAPA or IPMI (“no-CAPA/no-IPMI”). At 90 days, the overall survival between the three groups showed significant differences (Figure 2B). Pairwise comparisons revealed differences in survival rates between patients with IPMI and those with “CAPA only” or “no-CAPA/no-IPMI” (*p* = 0.005 and *p* = 0.002, respectively). There was no difference in outcomes between patients with “CAPA only” and those with “no-CAPA/no-IPMI” (*p* = 0.79).

Patients diagnosed with IPMI were more likely to receive *Aspergillus*-active antifungal therapy (8 out of 12) than patients with probable CAPA (8 out of 27) (*p* = 0.04) (Supplemental Table S2). Among patients with IPMI who received antifungal therapy, the survival rate was 37.5% (3 out of 8) at day 90. Conversely, patients with CAPA exhibited higher survival rates, regardless of whether they received active-mold antifungal therapy or not. Of the 8 patients with CAPA who received treatment, 4 (50.0%) were alive 90 days after ICU admission, while 8 of the 17 untreated patients (47.1%) survived 90 days after ICU admission (*p* = 1.00) (Supplemental Table S2).

## 4. Discussion

We analyzed the characteristics of patients who developed a fungal pulmonary superinfection after being admitted to the ICU for severe COVID-19, focusing on the differences according to the period of onset and the use of two different diagnostic criteria. This study included a substantial number of patients, providing the required power to address a certain number of questions. Our results suggest that pulmonary aspergillosis complications occur more frequently in immunocompromised patients: IPMI was observed in 7 of 30 (23.3%) patients meeting the EORTC/MSG immunosuppression criteria, compared with 5 of 217 (2.3%) immunocompetent patients.

In December 2020, we reported a low incidence of aspergillosis in patients with severe COVID-19 [7], in contrast to several case series reporting a very high incidence [2,5]. Our present work indicates that the cumulative incidence of aspergillosis either diagnosed by applying the ECMM/ISHAM criteria or through local assessment did not increase between the first and subsequent waves, particularly after the introduction of corticosteroid therapy, which therefore does not appear here as a risk factor for developing fungal complications. Other studies have investigated the epidemiological evolution of fungal infections during the various waves of the pandemic and provide divergent results. Fortarezza et al. found a significant increase in the incidence of mold infections between the first and second waves: 3% and 13%, respectively [20]. On the other hand, a single-center study showed an incidence of CAPA ranging from 4% to 6% with no significant difference during the waves [21], in line with our results.

Using our criteria, we determined the cumulative incidence of mold superinfection to be 4.8% (12/251). Using Firth’s penalized logistic regression, we found that IPMI was strongly associated with pre-existing host-related immunosuppression, particularly solid organ transplantation and long-term corticosteroid therapy. This is consistent with our previous results and with the data reported by Feys et al., who found that the presence of EORTC/MSG host factors among patients is associated with an increased incidence of CAPA [22].

As we noticed previously, the significant heterogeneity in the reported incidence of CAPA may be related to the criteria used to classify patients [23]. In our study, we used a local assessment system and compared it with the ECMM/ISHAM classification.

Consensus criteria are needed to provide a common framework for defining cases of invasive aspergillosis, especially for ICU patients [24,25]. We acknowledge it is a challenging effort to define consensus criteria, and it was not our intention to attempt to define other criteria. However, we had, with other authors, expressed some concerns about criteria defining CAPA because some arguments put forward in these consensus criteria were not supported by high-level evidence, such as histological analysis, as has been the case in other “pre-COVID-19” studies [26]. Moreover, the application of these recommendations did not retrieve the very high incidence of CAPA in the case series that were put forward to justify their own development [23]. An additional category, “Colonization”, could be added to the ECMM/ISHAM classification to identify patients whose diagnosis has not been clinically confirmed, as is already the case in certain algorithms [26]. Criteria such as an isolated positive result, a differential diagnosis, and a favorable outcome without treatment, etc., should be assessed to refine the classification.

By applying ECMM/ISHAM criteria, we found 33 patients with probable/possible CAPA, resulting in an overall cumulative incidence of 10.8% of probable CAPA and 2.4% of possible CAPA. Strikingly, most of these patients (47% of probable CAPA) did not receive any mold-active antifungal therapy, as the results of the mycological tests were considered insufficient to initiate targeted therapy, and were alive at D90. This suggests that the ECMM/ISHAM criteria for classifying cases as probable CAPA do not target invasive aspergillosis and likely include cases of non-invasive pulmonary aspergillosis or colonization or false-positive results in clinically asymptomatic patients who improve without antifungal therapy. This leads to a higher reported incidence of mold infection with the ECMM/ISHAM classification strategy.

The use of corticosteroid therapy in the management of severe COVID-19 has raised questions about an increased risk of infection, particularly about aspergillosis. The meta-analysis by Gioia et al. identifies corticosteroid therapy for COVID-19 as a risk factor for the development of CAPA [27]. However, the authors qualify their findings by pointing out that the detailed dose, frequency and duration of corticosteroid administration are not always specified in the included studies. Finally, they point out that in a subgroup of patients (classified as CAPA using strictly the ECMM/ISHAM criteria), corticosteroid therapy is no longer associated with the development of aspergillosis in COVID-19 patients. In univariate analysis, short-course corticosteroid therapy administered for COVID-19 showed a trend toward association with probable CAPA classification, but this association disappeared after adjustment for immunosuppression and BAL galactomannan sampling frequency in Firth’s penalized regression. Similarly, short-course corticosteroid therapy was not associated with IPMI in either univariate analysis or the sensitivity Firth model. In contrast, pre-existing long-term corticosteroid therapy remained associated with IPMI. These results support the need to distinguish short-course corticosteroids administered as standard-of-care treatment for severe COVID-19 from pre-existing corticosteroid-associated immunosuppression.

The analysis also showed that probable CAPA classification was associated not only with immunosuppression but also with BAL galactomannan sampling frequency. One can legitimately think that the more respiratory samples are taken, the more likely a pulmonary superinfection is to be diagnosed [28]. However, these results might also indicate that the increase in the number of BAL samples taken might result in an artificial increase in CAPA cumulative incidence by increasing the risks of false-positive mycological tests, leading to improper diagnosis. Indeed, many positive tests were not confirmed on further samples, and many patients improved without treatment. Gregoire et al. also reported a significant increase in the realization rate of BAL and of galactomannan research, *Aspergillus* PCR and *Aspergillus* cultures on BAL between the first and second waves of the pandemic. Interestingly, the authors reported that all CAPA cases (*n* = 9) were diagnosed during the second wave of the pandemic, and no significant difference in 250-day mortality was observed between CAPA and non-CAPA patients [29]. Unfortunately, most guidelines and recommendations do not sufficiently consider the possibility of false positives and the inconsistent specificity of galactomannan in a respiratory sample for the diagnosis of invasive aspergillosis [30,31].

The outcome analysis further supports the clinical relevance of distinguishing CAPA defined by ECMM/ISHAM criteria from IPMI retained by local multiparametric assessment. IPMI was associated with an important 90-day mortality rate, whereas patients classified as “probable CAPA only” (i.e., CAPA who were not also classified as IPMI with our local criteria) had a lower mortality rate. This gradient is consistent with the idea that ECMM/ISHAM criteria capture a broader group of patients, including some with uncertain clinical relevance, whereas the more stringent local assessment identifies a smaller subset of patients with more severe and prognostically meaningful mold infection. Nevertheless, these findings should not be interpreted as evidence of a causal effect of CAPA/IPMI on mortality. Overall mortality was analyzed in this study. CAPA/IPMI may act both as a direct contributor to poor outcomes and as a marker of greater clinical severity, prolonged ICU exposure, or more intensive diagnostic sampling. In addition, antifungal treatment was not randomly allocated and was more frequently administered to patients classified as IPMI, precluding any causal inference regarding treatment effect. Analyzing survival from ICU admission (rather than the date of infection) may introduce immortal time bias when comparing infected and non-infected patients. However, this bias is limited when comparing the CAPA and IPMI groups because the median time to infection onset is similar between these two groups in our cohort. Using a time-dependent Cox regression analysis, IPMI was significantly associated with higher 90-day mortality (*p* = 0.003), and probable CAPA showed a trend toward increased 90-day mortality (*p* = 0.074), although this association did not reach statistical significance. Nevertheless, the stronger association between IPMI and mortality supports the value of integrating mycological results with clinical course and repeated sampling rather than relying on isolated positive tests [24].

Our study has several limitations. First, it is monocentric, although it comprises five distinct and independent intensive care units, some of which have specific features (e.g., ECMO center). In fact, 63% of our patients are treated with ECMO because some of our units specialize in cardiovascular care. This rate is higher than what is reported in the literature on COVID-19 and fungal infection [24,32].

Second, our study does not include data on virus variants or assess the effect of vaccination, as vaccination was not yet available during most of the study period. During our study period (March 2020 to March 2021), France experienced successive waves of SARS-CoV-2 transmission. During 2020, corresponding to the first wave of our study, multiple viral introductions and subsequent dissemination of different viral lineages occurred. The D614G spike mutation, which emerged early during the pandemic, became predominant globally during the first half of 2020 [33,34]. However, systematic genomic surveillance had not yet been established, making it difficult to accurately determine the dynamics of variant circulation in Europe [35]. Genomic surveillance implemented from January 2021 documented the emergence and rapid spread of the Alpha (B1.1.7) variant in France, including in the Ile-de-France region, during the latter part of our study period. Although SARS-CoV-2 variants differed in transmissibility and disease severity over the study period, the similar mortality observed between the first and subsequent waves cannot be directly attributed to viral variants, as individual-level genomic data were not available. Changes in clinical management and supportive care over time may also have contributed to the observed outcomes. Furthermore, to establish a link between a SARS-CoV-2 variant and the onset of fungal complications, individual viral genomic data for each patient would be required (not available in our study); it would be ambitious to attempt to answer this question based solely on aggregate epidemiological data.

Our study covers only the period from March 2020 to March 2021; after March 2021, due to low cumulative incidence of aspergillosis at our center, screening of patients for fungal infections was no longer carried out systematically for all patients admitted with COVID-19.

Finally, the number of fungal infection events remained limited, particularly for IPMI. Although Firth’s penalized logistic regression was used to reduce small-sample bias and avoid overfitting, the models were intentionally restricted to a small number of prespecified covariates.

## 5. Conclusions

The cumulative incidence of SARS-CoV-2-associated invasive pulmonary mold infection remained stable over time. Solid organ transplantation and pre-existing long-term corticosteroid therapy were strongly associated with IPMI, whereas short-course corticosteroid therapy for COVID-19 was not. IPMI was associated with a high mortality rate, whereas patients classified as having probable CAPA had a lower mortality rate, similar to that of patients without fungal superinfection. Our findings underline that the classification system substantially influences both incidence estimates and outcome interpretation, and that probable CAPA classification may be partly influenced by diagnostic sampling intensity. Future studies that consider the limitations of diagnostic tests could help address the heterogeneity in reported incidences of these conditions.

## Figures and Tables

**Table 1:** Characteristics of the study population and comparison between the first COVID-19 epidemic wave and the subsequent waves.

	First Wave *n* = 171	Subsequent Waves *n* = 88	*p* Value *
Characteristics at ICU admission			
Age (years)	55 (46–64)	56 (48–64)	0.854
Sex, female	49/171 (28.7)	27/88 (30.7)	0.845
Active smoking	14/170 (8.2)	4/76 (5.3)	0.597
Hypertension	94/171 (55.0)	44/84 (52.4)	0.798
Diabetes	52/171 (30.4)	34/85 (40.0)	0.165
BMI > 25	113/165 (68.5)	61/81 (75.3)	0.339
COPD	11/171 (6.4)	3/84 (3.6)	0.559
HIV infection	6/171 (3.5)	2/83 (2.4)	1.00
Immunosuppression criteria	23/171 (13.5)	9/84 (10.7)	0.688
Hematological malignancy	5/171 (2.9)	3/84 (3.6)	0.721
Allogenic HSCT	2/171 (1.2)	1/84 (1.2)	1.00
SOT	14/171 (8.2)	4/84 (4.8)	0.437
Long-term corticosteroid therapy (>3 weeks, any dosage)	5/171 (2.9)	3/84 (3.6)	0.721
Severity criteria during ICU stay			
SAPS 2 at ICU admission	47 (33–65) (*n* = 133)	61 (53–70) (*n* = 65)	**<0.001**
Tracheal intubation	155/171 (90.6)	76/88 (86.4)	0.401
Intubation duration (days)	27 (14–45) (*n* = 148)	27 (16–43) (*n* = 63)	0.647
ECMO	88/159 (55.3)	63/81 (77.8)	**0.001**
Extra Renal Epuration	46/162 (28.4)	25/79 (31.6)	0.712
ICU stay length (days)	29 (15–50) (*n* = 161)	31 (20–54) (*n* = 82)	0.591
ICU outcome			
Death	66/163 (40.5)	36/85 (42.4)	0.879
Lost	8/163 (4.9)	3/85 (3.5)	
Specific COVID-19 therapy			
Hydroxychloroquine	62/165 (37.6)	0/82	**<0.0001**
Hydroxychloroquine + azithromycin	10/165 (6.1)	0/82	**0.033**
Lopinavir	30/165 (18.2)	1/82 (1.2)	**<0.0001**
Remdesivir	8/165 (4.8)	8/82 (9.8)	0.230
Tocilizumab	11/163 (6.7)	2/82 (2.4)	0.229
Short-term corticosteroid therapy	40/165 (24.2)	73/79 (92.4)	**<0.001**
(6 mg/day dexamethasone or 40 mg/day prednisone or equivalent)			

* Statistically significant *p* values are in bold. Categorical variables are presented as *n/N* (%), and continuous variables as median (IQR) (*n* = *N*). *N* represents the number of patients with available data for each variable; percentages are calculated using the corresponding available denominator. BMI, body mass index; COPD, chronic obstructive pulmonary disease; SOT, solid organ transplant; HSCT, hematopoietic stem cell transplant; SAPS, Simplified Acute Physiology Score; ECMO, extracorporeal membrane oxygenation; ICU, intensive care unit.

**Table 2:** Methods for mycological diagnosis and cumulative incidence of pulmonary mold infection according to two classification approaches in patients with severe COVID-19 during the first and subsequent waves of the pandemic.

	First Wave (*n* = 165)	Subsequent Waves (*n* = 86)	*p* Value *
Patients with ≥1 sample, *n* (%)			
Patient with at least 1 BAL	136 (82)	83 (96.5)	**0.001**
Patient with at least 1 serum	132 (80)	69 (80)	1
Samples per patient, median (IQR)			
Respiratory samples			
Respiratory tract sample culture ^1^	2.0 (1.0–5.0)	2.5 (1.0–4.0)	0.66
BAL culture	2.0 (1.0–4.0)	2.0 (1.0–5.0)	**0.012**
*A. fumigatus* specific PCR ^1^	2.0 (1.0–4.0)	2.0 (1.0–4.0)	0.35
BAL galactomannan	1.0 (1.0–4.0)	2.0 (1.0–4.0)	**0.0091**
Serum samples			
*A. fumigatus* specific PCR	0.0 (0.0–1.0)	0.0 (0.0–0.0)	**<0.0001**
Galactomannan	2.0 (1.0–5.0)	3.0 (1.0–7.0)	0.42
Beta-D-Glucan	2.0 (1.0–5.0)	3.0 (1.0–7.0)	0.24
Cases of invasive mold infection, *n* (%)			
According to local assessment (IPMI)	7 (4.2)	5 (5.8)	0.551
According to ECMM/ISHAM criteria (CAPA)	20 (12.1)	13 (15.1)	0.556
Possible CAPA	5 (3.0)	1 (1.2)	0.667
Probable CAPA	15 (9.1)	12 (14.0)	0.283

* Statistically significant *p* values are in bold. ^1^: all lower tract respiratory samples = BAL and Distal Protected Aspirate and Tracheal Aspiration (upper tract samples like sputum were not included). BAL, bronchoalveolar lavage; IPMI, invasive pulmonary mold infection; ECMM/ISHAM, European Confederation for Medical Mycology/International Society for Human and Animal Mycology; CAPA, COVID-19-Associated Pulmonary Aspergillosis; SD, standard deviation.

**Table 3:** Univariable comparisons and Firth’s penalized logistic regression of factors associated with probable COVID-19-Associated Pulmonary Aspergillosis (CAPA), as defined by the European Confederation of Medical Mycology (ECMM)/International Society for Human and Animal Mycology (ISHAM) consensus criteria. Possible CAPA (*n* = 6) are not included in this table.

	No CAPA (*n* = 218)	Probable CAPA (*n* = 27)	Univariate Analysis *p* Value *	Firth’s Penalized Logistic Regression OR (95%CI), *p* Value *
Characteristics at ICU admission				
First wave	145/218 (66.5)	15/27 (55.6)	0.26	
Age	56 (48–64)	53 (49–67)	0.35	
Female	67/218 (30.7)	6/27 (22.2)	0.36	
Active smoking	14/209 (6.7)	3/24 (12.5)	0.30	
Hypertension	113/214 (52.8)	16/27 (59.3)	0.53	
Diabetes	70/215 (32.6)	10/27 (37.0)	0.64	
BMI > 25	146/205 (71.2)	19/27 (70.4)	0.93	
COPD	14/214 (6.5)	0/27 (0.0)	0.17	
HIV infection	7/213 (3.3)	0/27 (0.0)	0.34	
Immunosuppression	22/214 (10.3)	7/27 (25.9)	**0.02**	**3.95 (1.39–10.64), 0.011**
Hematological disease	7/214 (3.3)	0/27 (0.0)	0.34	
Allogenic HSCT	3/214 (1.4)	0/27 (0.0)	0.54	
SOT	13/214 (6.1)	4/27 (14.8)	0.09	
Long-term corticosteroid ^1^	4/214 (1.9)	3/27 (11.1)	**0.007**	
Severity criteria during ICU stay				
Tracheal intubation	194/218 (89.0)	27/27 (100.0)	0.07	
Intubation duration (day)	27 (14–45)	37 (27–50)	0.34	
ECMO	124/201 (61.7)	21/27 (77.8)	0.10	
Extra Renal Epuration	55/202 (27.2)	11/27 (40.7)	0.15	
SAPS2 at ICU admission	53 (38–65)	66 (49–70)	**0.049**	
ICU stay length (day)	29 (18–50)	40 (33–66)	0.08	
Specific COVID-19 therapy				
Hydroxychloroquine	53/207 (25.6)	6/27 (22.2)	0.71	
Lopinavir	27/207 (13.0)	3/27 (11.1)	0.78	
Remdesivir	14/207 (6.8)	1/27 (3.7)	0.54	
Tocilizumab	13/208 (6.3)	2/26 (7.7)	0.62	
Corticosteroid therapy ^2^	92/203 (45.3)	17/27 (63.0)	0.09	1.65 (0.69–4.03), 0.259
Mycological data, median (IQR)				
Respiratory samples				
Respiratory tract sample culture ^3^	2.0 (1.0–4.0)	5.0 (2.0–7.0)	**0.0015**	
BAL culture	2.0 (1.0–4.0)	4.0 (2.0–7.0)	**0.0002**	
*A. fumigatus* specific PCR	2.0 (1.0–4.0)	4.0 (2.0–6.0)	**0.0046**	
BAL galactomannan	2.0 (1.0–4.0)	4.0 (2.0–6.0)	**0.0001**	**1.27 (1.11–1.48), <0.001**
Serum samples				
*A. fumigatus* specific PCR	0.0 (0.0–0.0)	0.0 (0.0–1.0)	**0.023**	
Serum galactomannan	2.0 (0.75–6.0)	5.0 (2.0–8.0)	**0.005**	
Beta-D-Glucan	2.0 (0.75–5.0)	5.0 (2.0–8.0)	**0.04**	

* Statistically significant *p* value is in bold. Categorical variables are presented as *n/N* (%), and continuous variables as median (IQR). *N* represents the number of patients with available data for each variable; percentages are calculated using the corresponding available denominator. ^1^ Long-term corticosteroid therapy: >3 weeks, any dosage. ^2^ Specific COVID-19 corticosteroid therapy: 6 mg/day dexamethasone or 40 mg/day prednisone or equivalent. ^3^ All lower tract respiratory samples = BAL and Distal Protected Aspirate and Tracheal Aspiration (upper tract samples like sputum were not included). BMI, body mass index; COPD, chronic obstructive pulmonary disease; SOT, solid organ transplant; HSCT, hematopoietic stem cell transplant; SAPS, Simplified Acute Physiology Score; ECMO, extracorporeal membrane oxygenation; ICU, intensive care unit; BAL, bronchoalveolar lavage.

**Table 4:** Univariable comparisons and Firth’s penalized logistic regression of host factors associated with invasive pulmonary mold infection (IPMI) according to the local clinical and mycological assessment.

	No IPMI (*n* = 239)	IPMI (*n* = 12)	Univariate Analysis *p* Value *	Firth’s Penalized Logistic Regression OR (95%CI), *p* Value *
Characteristics at ICU admission				
First wave	158/239 (66.1)	7/12 (58.3)	0.58	
Age	53 (42–61)	59 (49–67)	0.28	
Female	70/239 (29.3)	4/12 (33.3)	0.76	
Active smoking	17/230 (7.4)	1/9 (11.1)	0.67	
Hypertension	122/235 (51.9)	10/12 (83.3)	**0.03**	
Diabetes	76/236 (32.2)	5/12 (41.7)	0.50	
BMI > 25	159/226 (70.4)	10/12 (83.3)	0.34	
COPD	14/235 (6.0)	0/12 (0.0)	0.39	
HIV infection	7/234 (3.0)	0/12 (0.0)	0.54	
Immunosuppression criteria	23/235 (9.8)	7/12 (58.3)	**<0.0001**	
Hematological disease	7/235 (3.0)	0/12 (0.0)	0.55	
Allogenic HSCT	3/235 (1.3)	0/12 (0.0)	0.70	
SOT	13/235 (5.5)	5/12 (41.7)	**<0.0001**	**16.41 (4.37–62.50), *p* < 0.001**
Long-term corticosteroid ^1^	5/235 (2.1)	2/12 (16.7)	**0.003**	**18.31 (2.81–99.59), *p* = 0.004**
Severity criteria during ICU stay				
Tracheal intubation (yes/total)	213/239 (89.1)	12/12 (100.0)	0.23	
Intubation duration (day)	28 (15–45)	30 (10–47)	0.81	
ECMO	140/221 (63.3)	6/12 (50.0)	0.35	
Extra Renal Epuration	61/222 (27.5)	7/12 (58.3)	**0.02**	
SAPS2 at ICU admission	53 (39–66)	63 (50–88)	**0.03**	
ICU stay length (day)	30 (19–52)	35 (15–54)	0.92	
Specific COVID-19 therapy				
Hydroxychloroquine	56/227 (24.7)	5/12 (41.7)	0.19	
Lopinavir	29/227 (12.8)	1/11 (9.1)	0.65	
Remdesivir	15/227 (6.6)	0/12 (0.0)	0.84	
Tocilizumab	13/225 (5.8)	0/12 (0.0)	0.39	
Corticosteroid therapy ^2^	106/224 (47.3)	5/12 (41.7)	0.70	
Mycological data, median (IQR)				
Respiratory samples				
Respiratory tract sample culture ^3^	2.0 (1.0–5.0)	4 (1.75–6.25)	0.154	
BAL culture	2.0 (1.0–4.0)	4.0 (1.0–4.25)	0.298	
*A. fumigatus* specific PCR	2.0 (1.0–4.0)	3.0 (1.75–4.5)	0.201	
BAL galactomannan	2.0 (1.0–4.0)	4.0 (1.0–4.0)	0.210	
Serum samples				
*A. fumigatus* specific PCR	0.0 (0.0–0.0)	1.0 (0.75–2.0)	**<0.001**	
Serum galactomannan	3.0 (1.0–6.0)	3.5 (1.75–7.25)	0.212	
Beta-D-Glucan	2.0 (1.0–5.5)	4.0 (1.75–7.25)	0.167	

* Statistically significant *p* value is in bold. Categorical variables are presented as *n/N* (%), and continuous variables as median (IQR). *N* represents the number of patients with available data for each variable; percentages are calculated using the corresponding available denominator. ^1^ Long-term corticosteroid therapy: >3 weeks, any dosage. ^2^ Specific COVID-19 corticosteroid therapy: 6 mg/day dexamethasone or 40 mg/day prednisone or equivalent. ^3^ All lower tract respiratory samples = BAL and Distal Protected Aspirate and Tracheal Aspiration (upper tract samples like sputum were not included). BMI, body mass index; COPD, chronic obstructive pulmonary disease; SOT, solid organ transplant; HSCT, hematopoietic stem cell transplant; SAPS, Simplified Acute Physiology Score; ECMO, extracorporeal membrane oxygenation; ICU, intensive care unit; BAL, bronchoalveolar lavage.

## Data Availability

The original data presented in the study are openly available in Zenodo at https://doi.org/10.5281/zenodo.21977909.

## Supplementary Materials

The following supporting information can be downloaded at https://www.mdpi.com/article/10.3390/jof12090652/s1, Supplemental Table S1. Details of the criteria for defining a patient with invasive mold infection associated with COVID-19 according to the two classifications used in the study; Supplemental Table S2. Characteristics of patients with invasive mold infection during severe COVID-19 according to local assessment or ECMM/ISHAM CAPA assessment; Supplemental Table S3. Mycological data for all patients included who had at least one positive diagnostic test; Supplementary Figure S1. Exploratory principal component analysis of clinical and mycological sampling profiles according to CAPA/IPMI classification. (A) Projection of patients on the first two principal components according to final clinical classification (B) Main variables contributing to the first two principal components. PCA was performed using clinical severity, host-factor, and mycological sampling variables. CAPA/IPMI classification variables were not included as active variables. Missing values were handled by simple imputation, using median imputation for continuous variables and mode imputation for binary variables. PC1 and PC2 explained 37.1% and 12.9% of total variance, respectively.
