# Supplementary material for: Invasive Aspergillosis Complicating Severe COVID-19: Cumulative Incidence, Risk Factors, and Outcomes Between the First Wave of the Pandemic and Subsequent Waves, According to the ECMM/ISHAM Classification and a Local Case Assessment
Source: J Fungi (Basel). 2026 Sep 1;12(9):652. doi: 10.3390/jof12090652 (PMC13608678; doi:10.3390/jof12090652)
Supplement: Supplementary file 1 [file jof-12-00652-s001.zip › jof-4476029 Supplemental Table S2 Invasive aspergillosis complicating severe COVID-19.pdf]

Supplemental Table S2: Characteristics of patients with invasive mold infection during severe COVID-19 according to local assessment or ECMM/ISHAM CAPA assessment.

	IPMI (Local assessment) n = 12 patients	Probable CAPA (ECMM/ISHAM) n = 27 patients	p-value
Characteristic at the ICU's admission			
Age (year)	59 [49-67]	53 [49-67]	0.164
Male	4 (33.3)	6 (22.2)	0.692
Active smoking	1 (11.1)	3 (12.5)	0.999
Hypertension	10 (83.3)	16 (59.3)	0.279
Diabetes	5 (41.7)	10 (37.0)	0.999
BMI > 25	10 (83.3)	19 (70.4)	0.692
COPD	0 (0.0)	0 (0.0)	0.999
HIV infection	0 (0.0)	0 (0.0)	0.999
Immunosuppression criteria	7 (58.3)	7 (25.9)	0.074
Hematological disease	0 (0.0)	0 (0.0)	0.999
Allogenic HSCT	0 (0.0)	0 (0.0)	0.999
SOT	5 (41.7)	4 (14.8)	0.101
Long term corticosteroid therapy (> 3 weeks, any dosage)	2 (16.7)	3 (11.1)	0.634
Severity criteria during ICU stay			
Tracheal intubation	12 (100)	27 (100)	0.999
Intubation duration (day)	30 [10-47]	37 [27-50]	0.356
ECMO	6 (50.0)	21 (77.8)	0.133
Extra Renal Epuration	7 (58.3)	11 (40.7)	0.487
Noradrenaline >0.5mg/h	10 (83.3)	21 (77.8)	0.999
Prone position	11 (91.7)	24 (88.9)	0.999
SAPS2 (median)	63 [50-88]	66 [49-70]	0.437
ICU stay (day)	35 [15-54]	40 [33-66]	0.188
ICU death			
Yes	9 (75.0)	13 (48.1)	0.168
Lost	1 (8.3)	2 (7.4)	0.999
Specific COVID-19 therapy			
Hydroxychloroquine	5 (41.7)	6 (22.2)	0.261
Hydroxychloroquine + azithromycin	0 (0.0)	1 (3.7)	0.999
Lopinavir	1 (8.3)	3 (11.1)	0.999
Remdesivir	0 (0.0)	1 (3.7)	0.999
Tocilizumab	0 (0.0)	2 (7.7)	0.999
Corticosteroids > 40mg prednisone	5 (41.7)	17 (63.0)	0.299
Characteristics of mold infections			
Time of infection (day)	13 [8-20]	16 [9-23.5]	0.604
Antifungal treatment [1]	8 (66.7)	8 (29.6)	**0.040**
Treated and alive at D90	3/8 (37.5)	4/8 (50)	1.000
Untreated and alive at D90	0/4 (0.0)	8/17 (47.1)	0.131
Mycological data (mean ± SD)			
**<u>Respiratory samples</u>**			
Respiratory tract sample culture	4.5 ± 3.2	5.0 ± 3.2	0.655
BAL culture	3.7 ± 3.1	4.7 ± 3.2	0.369
A. fumigatus specific PCR	3.7 ± 2.6	4.1 ± 2.6	0.660
BAL galactomannan	3.5 ± 3.0	4.4 ± 2.9	0.381
**<u>Serum samples</u>**			
A. fumigatus specific PCR	1.3 ± 1.0	0.6 ± 0.8	**0.025**
Serum galactomannan	4.8 ± 4.0	5.3 ± 3.6	0.700
Beta-D-Glucan	4.8 ± 3.8	5.3 ± 3.6	0.696

Statistically significant value is in bold.
Categorical variables are presented as n (%) or n/N (%) if there are missing data.
Continuous variables as median [IQR] (N), unless otherwise indicated.
Mycological results are presented as mean ± Standard Deviation.

[1] Antifungal treatment: effective against molds infections (azoles such as voriconazole or isavuconazole; deoxycholate or liposomal amphotericin B)

BAL, bronchoalveolar lavage; CAPA, COVID-19 associated pulmonary aspergillosis; ECMM/ISHAM, European Confederation for Medical Mycology / International Society for Human and Animal Mycology, BMI, Body mass index; COPD, Chronic obstructive pulmonary disease; SOT, Solid organ transplant; HSCT, Hematopoietic stem cell transplant; SAPS, Simplified Acute Physiology Score; ECMO, Extracorporeal membrane oxygenation; SD, standard deviation
